# Pneumorrhachis After an Upper Respiratory Infection: A Case Report of a Rare Phenomenon

**DOI:** 10.7759/cureus.7784

**Published:** 2020-04-22

**Authors:** Killian Llewellyn, Ryan Johnson, Evan M Krueger, Jason M Seibly

**Affiliations:** 1 Surgery, Midwestern University Chicago College of Osteopathic Medicine, Downers Grove, USA; 2 Neurosurgery, Advocate BroMenn Medical Center, Normal, USA; 3 Neurosurgery, Advocate Health Care, Downers Grove, USA; 4 Neurosurgery, Central Illinois Neuroscience Foundation, Bloomington, USA

**Keywords:** pneumorrhachis, intraspinal air

## Abstract

Pneumorrhachis (PR) is the presence of free air within the spinal canal. It is generally benign and improves with conservative management. Case reports and a literature review exist documenting the existence and potential pathogenesis of this phenomenon, but no evidence-based guidelines exist documenting what treatment, if any, is indicated for this condition. We present a case of a 21-year-old male who developed PR after a preceding upper respiratory tract infection. His symptoms improved with expectant management and administration of high-flow oxygen. The purpose of this case report is to add to the scarce existing literature reporting this condition and to provide a short review of literature detailing the pathogenesis of PR.

## Introduction

Pneumorrhachis (PR) is a rare, typically benign condition of air within the spinal canal resulting from iatrogenic, traumatic, or non-traumatic causes. Evidence-based guidelines for the management of this condition are lacking; therefore, treatment for PR is documented through various case reports. The etiology of PR can often be helpful in determining the appropriate course of treatment. In a majority of cases, conservative management with attention paid to cardiorespiratory support and neurologic monitoring is indicated. Surgical intervention is entertained for PR in the situations causing neurologic deterioration and cerebrospinal fluid leaks, or in situations that are secondary to fistulous connections from extra-spinal sources [1]. In this case report, we present a case of a 21-year-old patient with the rare finding of PR after a recent upper respiratory infection.

## Case presentation

**Figure 1:**
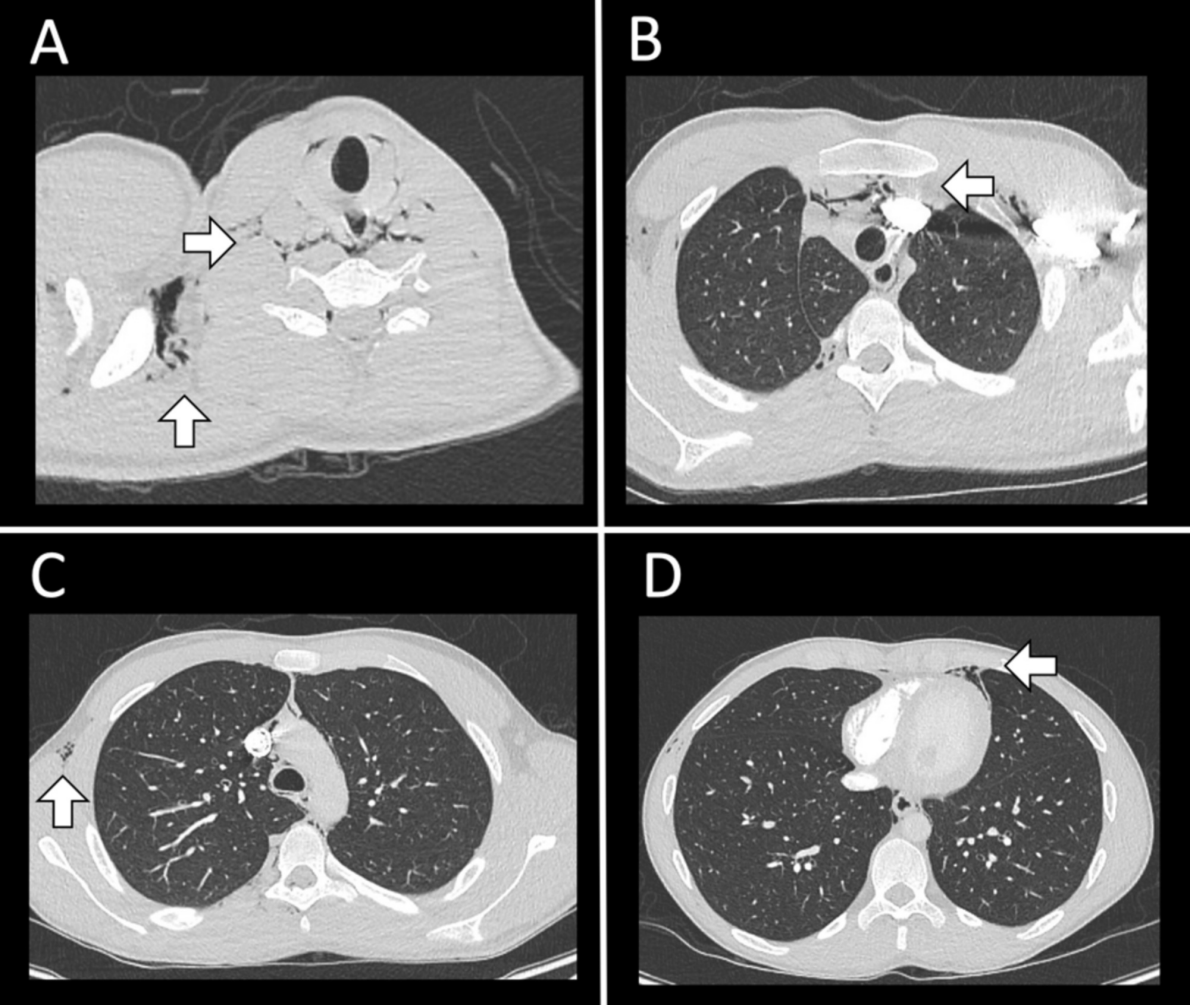
Computed tomography (CT) chest scan upon presentation with non-productive cough and sternal region chest pain. (A-D) Axial slices showing scattered pneumomediastinum with extension into the supraclavicular neck, bilateral axilla, and right-greater-than left chest wall. There is no obvious pleural fistula. There is a metal artifact from the previous left clavicular hardware.

**Figure 2:**
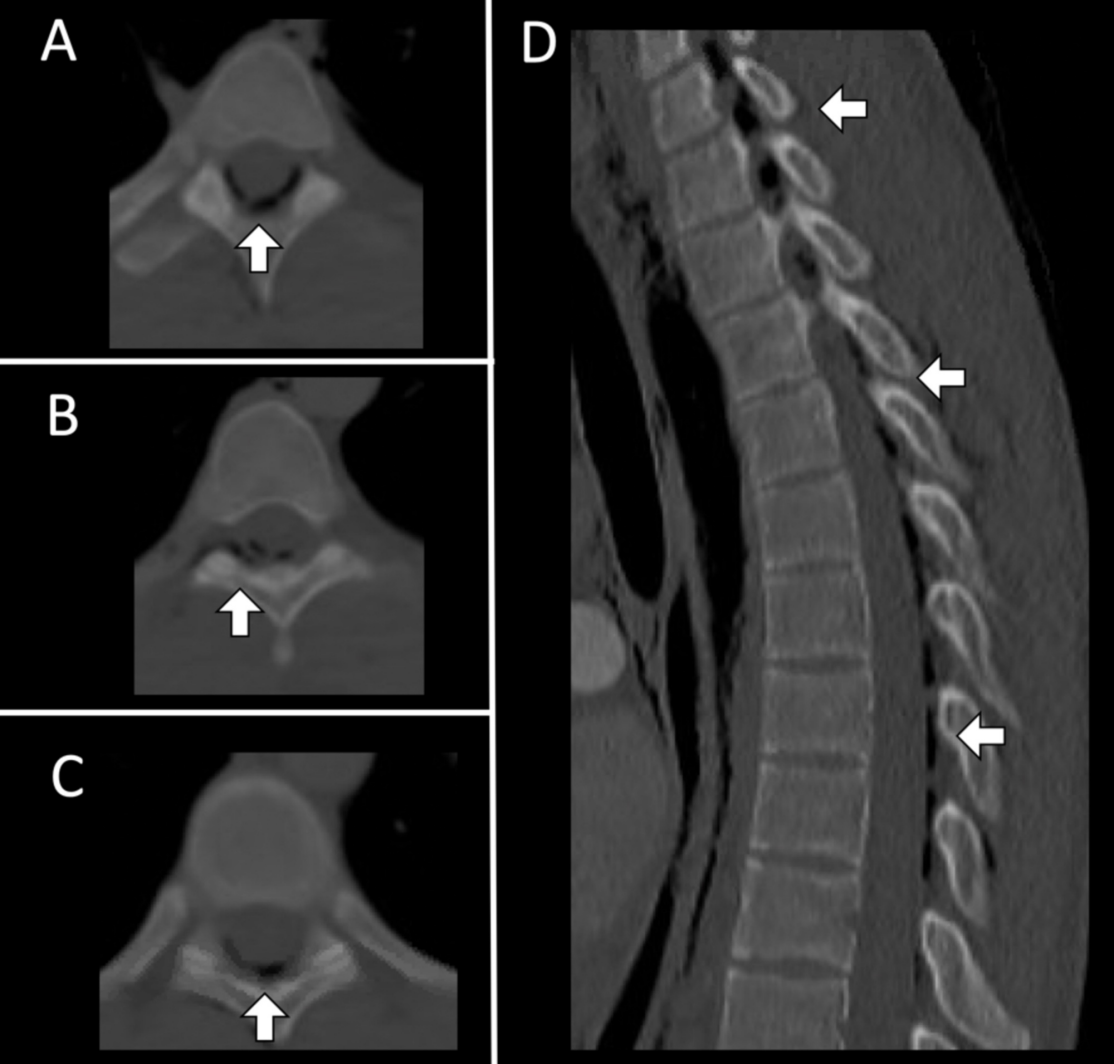
Computed tomography (CT) scan of the thoracic spine upon presentation with non-productive cough and sternal region chest pain. (A-C) Axial and (D) sagittal sequences showing scattered extradural pneumorrhachis throughout the central thoracic canal and right-greater-than left chest wall.

**Figure 3:**
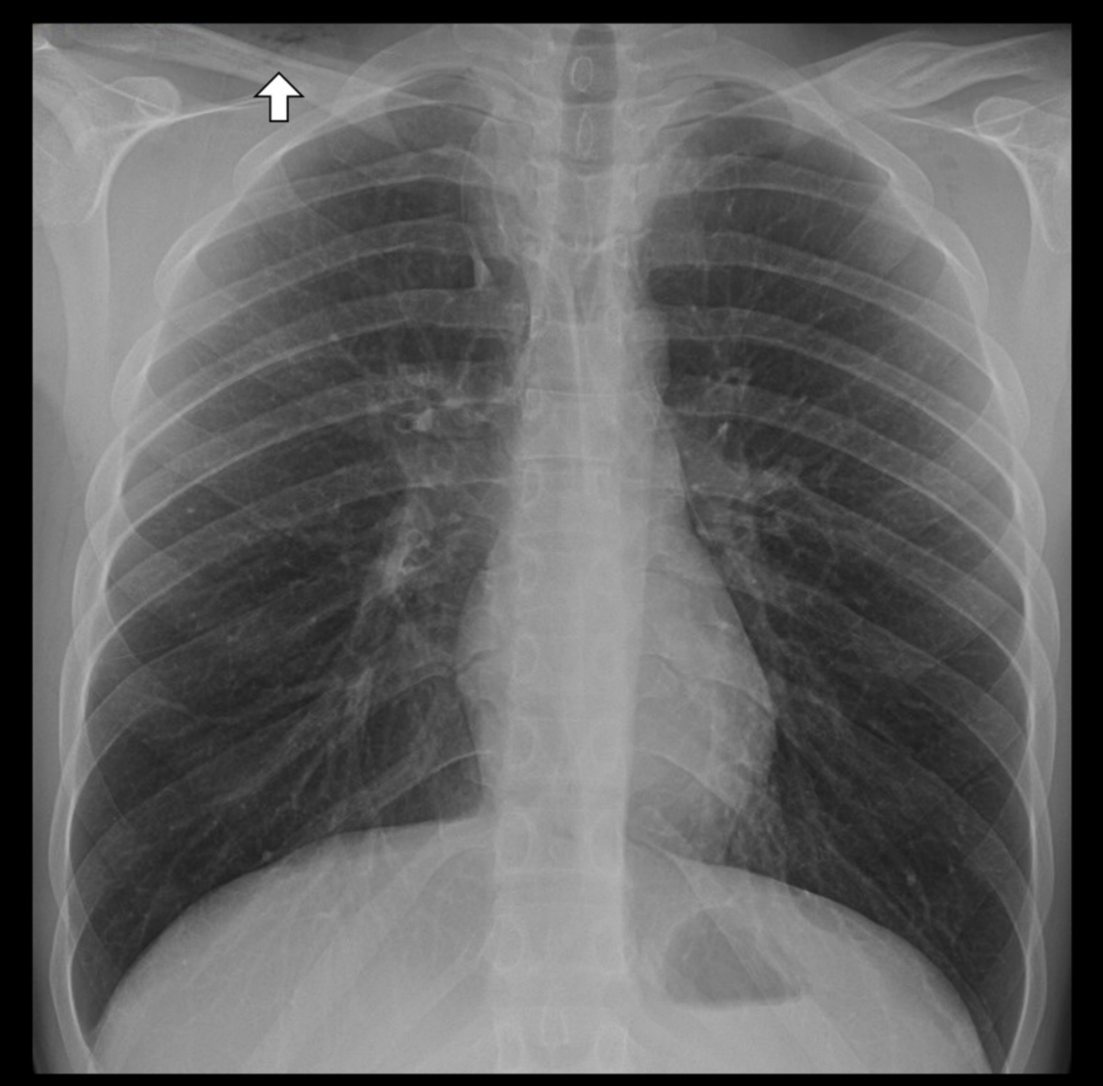
Plain film chest X-ray 12 hours after treatment with 12 liters per minute oxygen through a non-rebreather. Persistent pneumomediastinum with decreased soft tissue emphysema after treatment with high-flow oxygen.

A 21-year-old male presented with acute onset chest pain following a one-week history of rhinorrhea, nasal congestion, and a non-productive cough. Initial chest radiograph demonstrated linear lucencies in the mediastinum and right supraclavicular region suggestive of pneumomediastinum. A subsequent computed tomography angiogram (CTA) of the chest was obtained, which showed pneumomediastinum with subcutaneous emphysema involving the supraclavicular lower neck region, bilateral axilla, right thoracic paraspinal musculature, and the right chest wall (Figure 1). Additionally, the CTA of the chest demonstrated hypoattenuation within the thoracic spinal canal from the sixth cervical vertebra to the ninth thoracic vertebra, consistent with PR (Figure 2). His neurological examination was unremarkable for focal deficits or signs of myelopathy. Neurosurgical intervention was not recommended. The patient was started on 12 liters per minute of oxygen through a non-rebreather mask for 12 hours and was admitted for observation. On hospital day 1, a repeat chest radiograph demonstrated persistent pneumomediastinum with decreased soft tissue emphysema (Figure 3). The patient was discharged home on hospital day 2, neurologically intact.

## Discussion

PR is defined by the presence of air in the spinal canal. It is a rare condition that is only sporadically reported in the literature. This condition has previously been referred to as intraspinal pneumocele, spinal epidural emphysema, and aerorachia, until the current term of PR was coined by Newbold et al. in 1987 [2]. PR can occur traumatically, iatrogenically, or spontaneously [1]. Oertel et al. in 2006 published a comprehensive review of literature documenting 86 cases of this epiphenomenon. In their paper, they documented 13 reported cases of PR due to violent coughing from an upper respiratory infection or asthma, similar to that in our patient [1,3]. Mechanisms for intraspinal air entry from a respiratory source have been previously described to involve a one-way air valve mechanism from ruptured alveoli. The pressure gradient created between the intra-alveolar space and the perivascular interstitium allows air to enter the interstitial tissues surrounding the mediastinum. Subsequent extension through paraspinal fascial planes and nerve root sheaths within the neural foramina allow access to the epidural space [1,2]. Additionally, Tsuji et al. specifically described the potential for high-pressure gradients from violent coughing, separating the mediastinal pleura from bilateral parietal pleurae, thus permitting air to travel dorsally toward the vertebral column and spinal canal [4].

PR is generally asymptomatic, but it can present with neurological deficits in the event of air compressing the spinal cord [1,5-6]. While there are no evidence-based guidelines for the treatment of PR, various case reports have documented that PR can be managed conservatively depending on the etiology. There is a theoretical benefit of introducing high-flow oxygen as it can increase the oxygen content of the entrapped gas, leading to more rapid absorption. In the similar phenomenon of pneumocephalus, which is defined by the presence of intracranial air, Hong et al. demonstrated there was an increased rate of air reabsorption in those receiving 100% oxygen compared with room air [7]. Surgical management for PR is often considered in the setting cerebrospinal fluid leaks, fistulous connections from extra-spinal sources, or myeloradiculopathy resulting from tension PR [1].

Our case illustrates the occurrence of PR without any associated macro-trauma. The patient’s recent upper respiratory infection preceding admission potentially instilled a high-pressure micro-trauma to his alveoli during coughing events, leading to air escaping in the mechanism described above. No neurological deficits were observed on physical examination, which prompted conservative management. The patient’s symptoms improved during his hospitalization with expectant management and administration of high-flow oxygen. Further imaging would be needed to determine if the PR resolved; however, this would require additional risk associated with radiation that would outweigh the potential benefit in light of the patient’s improving condition.

## Conclusions

PR is a rare and likely under-diagnosed phenomenon. The underlying etiology and physical examination are critical for management consideration, but this condition is typically treated conservatively. A single-center, large-volume case series would be helpful in guiding management decisions.
